# A cross-sectional and semantic investigation of self-rated health in the northern Sweden MONICA-study

**DOI:** 10.1186/1471-2288-12-154

**Published:** 2012-10-09

**Authors:** Göran Waller, Peder Thalén, Urban Janlert, Katarina Hamberg, Annika Forssén

**Affiliations:** 1 Department of Public Health and Clinical Medicine, Division of Family Medicine, Umeå University, Umeå, Sweden; 2 Department for Cultural Studies, Religious Studies and Educational Sciences, University of Gävle, Gävle, Sweden; 3 Department of Public Health and Clinical Medicine, Division of Epidemiology and Global Health, Umeå University, Umeå, Sweden

## Abstract

**Background:**

Self-Rated Health (SRH) correlates with risk of illness and death. But how are different questions of SRH to be interpreted? Does it matter whether one asks: “How would you assess your general state of health?”(General SRH) or “How would you assess your general state of health compared to persons of your own age?”(Comparative SRH)? Does the context in a questionnaire affect the answers? The aim of this paper is to examine the meaning of two questions on self-rated health, the statistical distribution of the answers, and whether the context of the question in a questionnaire affects the answers.

**Methods:**

Statistical and semantic methodologies were used to analyse the answers of two different SRH questions in a cross-sectional survey, the MONICA-project of northern Sweden.

**Results:**

The answers from 3504 persons were analysed. The statistical distributions of answers differed. The most common answer to the General SRH was “good”, while the most common answer to the Comparative SRH was “similar”. The semantic analysis showed that what is assessed in SRH is not health in a medical and lexical sense but fields of association connected to health, for example health behaviour, functional ability, youth, looks, way of life. The meaning and function of the two questions differ – mainly due to the comparing reference in Comparative SRH. The context in the questionnaire may have affected the statistics.

**Conclusions:**

Health is primarily assessed in terms of its sense-relations (associations) and Comparative SRH and General SRH contain different information on SRH. Comparative SRH is semantically more distinct. The context of the questions in a questionnaire may affect the way self-rated health questions are answered. Comparative SRH should not be eliminated from use in questionnaires. Its usefulness in clinical encounters should be investigated.

## Background

When questions about self-rated health (SRH) were first included in questionnaires, this was done in parallel to the social greeting “how are you?” This implied a conversational way of introducing questions about health matters, intended to form a relationship and show interest and care [1]. When correlations with premature death, stroke, depression, and functional capacity proved stronger than either a doctor’s medical judgement or biomedical indicators, the function in the scientific context was changed [1,2]. The answers tended to become treated as propositional statements and having the more or less objective function of reporting the respondent’s actual health condition.

However, this simplistic, straightforward interpretation has subsequently been revised. The answers to SRH questions correlate with life habits, disease, physical functional ability, symptoms, education, income, wealth, social capital, age, sex, parental health, attitudes etc [3-9]. Interpretation of SRH has been complicated by the fact that neither subjective health (personal assessment of health) nor objective health (health as regarded in medical theory and practice) has a generally accepted definition. Consequently the measure is unspecific. How do you interpret a measure when you do not know what it refers to?

Two alternative formulations are often used in questionnaires. These could take the form: “How is your health in general?” (General SRH-question) and “How would you judge your health compared to other people of your age?” (Comparative SRH-question) [10]. It has been claimed that the wording of the question has little significance, and that the answers to all different formulations of the question represent parallel judgements of SRH [1,4]. However Baron-Epel et al. and Sargent-Cox et al. have argued that the two ways of asking shed light over different aspects of SRH [11,12]. Little attention has been paid to the potential importance of the context surrounding the questions in a questionnaire, such as the questions preceding the questions investigated. This could influence the way SRH questions are interpreted, and hence the answers. One way to increase our understanding of SRH-questions as a measure would be to analyse the questions semantically, to find out how the questions might function and to reveal possible differences between the General and Compared SRH-questions. In this study we therefore investigate two alternative formulations of the SRH question, one general and one comparative, using semantic analysis of the wording and statistical analysis of the outcome from a cross-sectional survey. We also investigate differences in numerical outcomes related to different contexts surrounding the questions in a questionnaire.

Our research questions are: 1) Are their differences in the statistical distribution of the answers to the two formulations of the question? 2) What are the semantic meanings of a General SRH-question and of a Comparative SRH-question, and how do these meanings differ? 3) Does the context for the question influence the answers given?

## Methods

Questionnaires from the MONICA project (Multinational Monitoring of Trends and Determinants in Cardiovascular Disease) in North Sweden issued in 1990, 1994, 1999, were investigated [13]. The MONICA project included a risk-factor analysis, with questions concerning education, hospitalisation for heart infarction, having suffered a stroke, and diagnosis for diabetes. Participants were selected randomly from the population register of the two most northerly regions of Sweden and sorted by sex and age. In 1990 they were 25 to 64 years old; in 1994 and 1999 they were up to 74 years old.

The questionnaire was divided into two parts. Part 1 was answered at home and returned by post. Part 2 was answered in combination with a medical investigation and sample testing at the nearest health care unit. Forty units were involved. Two questions on self-rated health were included. In 1990 and 1994, these were worded:” How would you assess your general health condition – good, bad, or somewhere in between” (General SRH) and” How would you assess your general health condition compared with other persons of the same age - better, worse, similar?” (Comparative SRH). Since answers to both SRH questions were separated into three alternatives for those years, they could be used without regrouping the primary data. The response patterns were analysed statistically. A frequency calculation was carried out on answers to the SRH questions, and the Spearman Rank-ordered correlation coefficient calculated using SPSS18.

The answers from questionnaires in 1994 and 1999 were used to study the influence of a different context for the Comparative SRH question, since this had been changed in the meantime. The General SRH question was not analysed as it was reformulated in 1999 to a five-grade scale making comparison with previous years less meaningful. Statistical significance was estimated with the Chi-2 test.

For the semantic analysis a basic semantic methodology was employed [14]. Semantics is a method for understanding the meaning of words and sentences. The overriding philosophical framework is influenced by Ludwig Wittgenstein’s theory of language as expressed in his later works [15]. He used the term “language-game”, and gave examples to demonstrate that a words’ significance depends on how it is used, and in what context. In this way, Wittgenstein asserted that the relationship of a word to an underlying reference is not fixed but depends on social and cultural practice at that time. The analysis performed involved: the social and practical context for the questionnaire; similarities and differences between the questions depending on their principal words; the context for the actual questions in the questionnaire.

The investigation was carried out within the approval for the Northern Sweden MONICA-study by the Research Ethics Committee of Umeå University. All subjects gave informed consent to participate.

## Results

### Statistical analysis

There were 1583 responses to the MONICA questionnaire in 1990, and 1921 responses in 1994. The response rates were 79% and 77% respectively. Table 1 gives the respondents’ age and sex distribution, together with the incidence of self-reported diabetes, heart infarction, and stroke. Forty-two percent of participants were 25-44 years old, 47% were aged 45-64, while 11% were 65 or older. Comparing the assessments made by all participants, 62% rated their General Health as “good”, 2% as “bad”, and 36% as “somewhere in between” (not shown in table). Fourteen percent rated their Comparative Health as “better”, 12% as “worse”, and 74% as “similar”. The Spearman rank-ordered correlation between General and Comparative SRH was 0.48.

**Table 1:** Distribution of answers on General self-rated health and Comparative self-rated health for men and women in relation to age-group

	**Age-group**		
	**25-44 years n = 711**	**45-64 years n = 813**	**65 years and older n = 193**
***Men: *n = 1717**			
General self-rated health	%	%	%
Good	70.9	56.9	50.3
In between	26.4	39.1	46.1
Bad	2.1	3.4	2.6
Compared self-rated health			
Better	14.1	17.6	23.8
Similar	76.2	66.5	65.3
Worse	9.0	15.3	9.8
Spearman r	0,47	0,54	0,55
Share with self-reported disease	1.3	10.0	26.4
***Women: *n = 1787**			
General self-rated health	%	%	%
Good	70.6	56.4	45.9
In between	27.2	41.0	52.1
Bad	1.7	2.0	1,5
Compared self-rated health			
Better	7.5	12.8	17.0
Similar	82.1	73.9	68.0
Worse	10.2	11.6	13.9
Spearman r	0,50	0,46	0,47
Share with self-reported disease	1.2	4.8	11.3

Share with self-reported disease (stroke, diabetes, myocardial infarction) as percent of age-group.

Table 1 compares the distribution of responses to the two questions for men and women in the three age groups. General SRH falls off with increasing age. By contrast, Comparative SRH shows an increasing number of “better” replies with increasing age. The answers to the General SRH question are skew distributed, with only 1 - 3% selecting the alternative “bad”. The answers to the Comparative SRH question show a more normally distributed response pattern. Men and women give similar estimates for their general health, while more men than women estimate their comparative health as “better”. A greater proportion of women choose the “similar” alternative. A Chi-2-test was used comparing all men to all women with the answers to Comparative SRH as outcome (Chi-2 26.51 and df2, p < 0,001).

**Figure 1:**
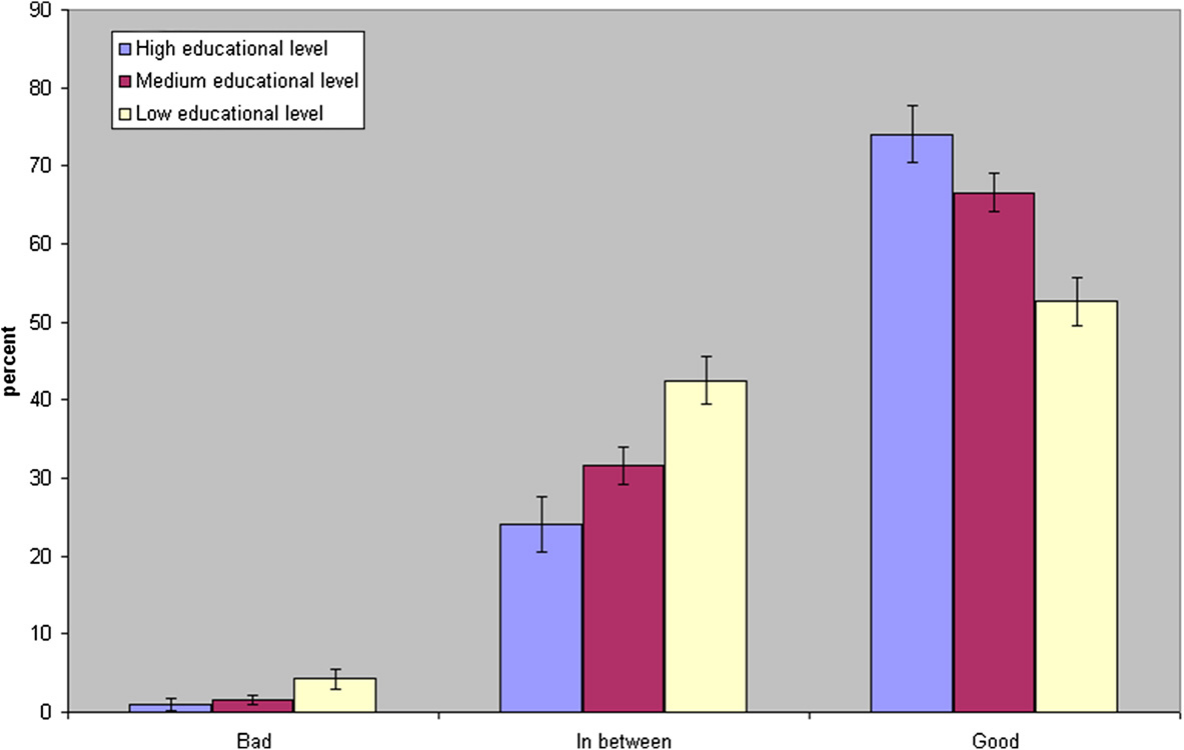
**General self-rated health in relation to educational level in men and women aged 25-64 years in 1990 and 1994.** n = 3086. Confidence interval 95%.

**Figure 2:**
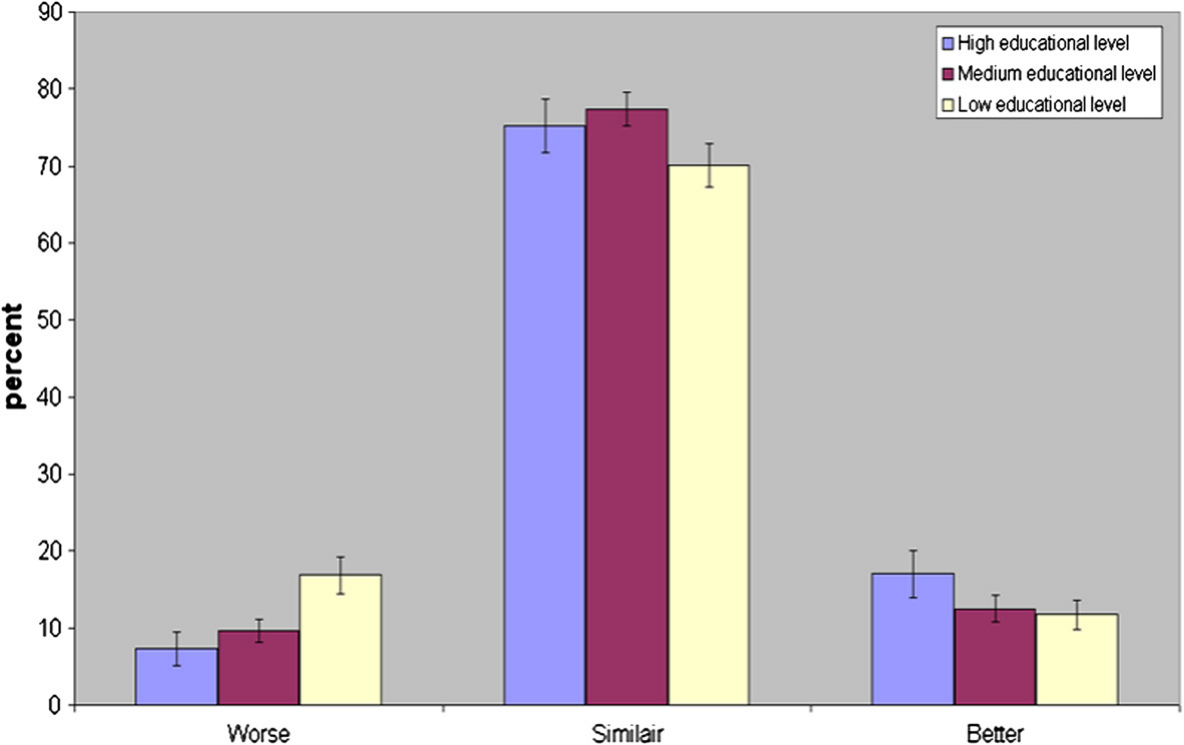
**Comparative self-rated health in relation to educational level in men and women aged 25-64 years in 1990 and 1994.** n = 3077. Confidence interval 95%.

Figure 1 shows how education correlated to General SRH for persons 25-64 years. The answer “good” is significantly more common from the highly educated than from the medium and low educated. Figure 2 shows how education correlated to Comparative SRH for persons 25-64 years. Most obvious is the difference in educational level among those assessing their health condition as “worse”. Less educated persons age 45-64 gave the alternative “worse” in 17% of cases, while well-educated 45-64-aged persons gave the answer “worse” in only 7% of cases (not shown in figure, 95% CI for difference 6%-14%; p < 0.001). Men and women aged 25-44 showed a similar pattern.

The response distribution to the Comparative SRH question changed significantly between 1994 and 1999. In 1994, 1921 persons answered while in 1999, 1823 persons answered. They were aged 25-74 years and selected in the same manner in 1994 and 1999. The response rates were 77% and 73% in 1994 and 1999 respectively. The proportion selecting the alternative “better” increased from 15% to 22%, i.e. a 7 percent units increase, between the two years (Chi-2-test 41.0 df2, p < 0.001).

### Semantic analysis

#### Social and practical context

The chosen regions of North Sweden were homogeneous from a linguistic and cultural perspective. Economic, social, and educational differences were small [[16] (pp 110, 174)]. There were few immigrants. The investigation was carried out for a research project that had attracted much attention in the mass media. It was therefore well known to the local population. Its medical character was tied to the local health-care system. The project was an invitation to participate in an event of wider importance.

#### Semantics of general SRH

The question “How would you assess your general health condition – good; bad; somewhere in between?” has as principal words the verb “assess”, and the noun “health” with the adjective “general”. What do these words denote, i.e. what are the dictionary definitions of the words and what are their sense-relations [14]. The term “sense-relations” gives a broader understanding of the word, its relationship with other words, linguistic expressions, and area of application. From the dictionary, the word “assess” means “to determine the amount or value of something” or, more neutrally, “value”. The sense-relations and historic applications of the word are described in more comprehensive dictionaries. It is here used as a request to make a careful evaluation of a situation or circumstance (see Table 2). The word “health” is not clearly defined. According to one dictionary it means “state of well-being, state of being vigorous and free from disease”. The sense-relations are significantly wider than that, connecting to functional capability, habits, youth, appearance, lifestyle, sickness, medical treatment, diet etc. In the same dictionary, the word “general” is defined as “what can only be characterised in general terms, covering the totality while ignoring the details”. The sense-relations coupled to “general” are extremely wide. The effect on meaning of combining the adjective “general” with “health” is therefore an additional emphasis on not being specific. For an overview, see Table 2. How the adjectives “good”, “bad” and the adverb “in between” are used in the population and by the person answering the question affects the answers. Adjectives in valuations could be interpreted as propositional statements as well as expressions of feelings and attitudes. The use of adjectives makes the answers ambiguous.

**Table 2:** Main words and examples of their denotations and sense-relations

**Primary word**	**Examples of denotation**	**Examples of sense relations**
Assess	Determine the amount or value of something	Legal assessment
	Make a valuation	Somewhat well considered valuation
		Competition
		Judgement
		Certificate
		Count points
		Place on a scale etc.
Health	State of well-being, freedom from disease with full bodily functioning	Functioning capability
		Youth
		Beauty
		Habits, lifestyle, diet
		Exercise, gym, advertising
		claims etc.
General	Concerning all, or almost all	Comprehensive
	On the whole	Vague
	Ignoring details	Imprecise
	Unspecific	Uncertain
		Abstract

#### Semantics of comparative SRH

The question”How would you assess your general health condition compared with other persons of the same age - better, worse, similar?” has most of its principal words in common with the General SRH question. The word “compared” is one essential difference between the two sentences. The word “compare” has the denotation of assessing (something) in relation to (something else) to find the similarities or differences. Its sense-relations are coupled to words like grading, order, assessing, and classifying. The word “persons” directs attention to individuals, not an anonymous group of people. The direction to compare with other people of one’s own age leads to domains of significance such as social comparison, performance, profession, income, and achievement in life. Such comparisons are coupled to ranking, and the creation of hierarchies and value structures. Comparisons are used frequently in both everyday speech and writing. Comparative SRH provides the respondents with a reference system, namely “compared with other persons of the same age”. This gives the answers a more determinate character and provides less room for purely subjective evaluation. The alternative replies to the Comparative SRH question consist of the comparing adjectives, “better”, “worse” and “similar”. Thus, the answer is influenced by the double challenge to compare, firstly what is given by the question itself, and secondly by the alternatives provided.

#### Context for the questionnaire

In 1994, the SRH questions were placed in the section that was sent out by post and answered at home. The SRH questions followed each other in the questionnaire, and were placed in a context concerning diseases and cardiovascular mortality of close relatives. A drawing implying old grandparents accompanied the questions. In 1999, the SRH questions were moved to the section that was completed in combination with the visit to the health care unit for medical investigation. The drawing of grandparents was removed and the SRH questions were opening questions, implying that the other questions – which might have reminded the respondents of negative health outcomes or hereditary disease in the family - had less influence on how they were understood and answered.

## Discussion

### Summarizing answers to the research questions

Statistical partitioning between the alternative answers differed significantly for the two questions. The study replicated already known differences in answers to General and Comparative SRH. The General SRH question did not imply comparison whereas the Comparative SRH question did, both directly and by the answering alternatives given. The comparison involved “sense-relations” coupled to the word “health”. The word “persons” steered the question towards concrete comparisons. Both words might, in this context, imply a comparison involving a stratification of social status, which are well-known to influence health [17]. The answers to the General SRH question depended on the way the adjectives (bad; in-between; good) were used, whereas the answers to the Comparative SRH question depended on a consideration of own health in relation to other persons.

The changed response distribution between 1994 and 1999 to the Comparative SRH question indicates that the answers might have been influenced by the considerable change in the context that had taken place between those years.

#### Strengths and weaknesses of this study

The study was based on good quality data. The data could be processed without recoding alternative answers, and connects directly with the choices made by participants. This helps in understanding how the questionnaire functioned for the participants. The semantic method was based on well-established principles. The validity of the analysis must be judged with criteria appropriate for the method, namely proper use of concepts, logical consistency, internal coherence and plausibility. Our conclusion about a semantic difference between the General and Comparative SRH questions was corroborated by statistical analysis of the answer distribution. In addition, the semantic analysis provided an explanation of the numerical outcome, which further empirical investigations would not have yielded. That the outcome of the Comparative SRH question changed between 1994 and 1999 also satisfies semantic theories about the influence of situation and context for the interpretation and function of utterances and questions.

Semantic theory is applicable to all natural languages. Therefore we argue that the semantic method can be generalised to other languages and cultures. However, the numerical distribution of answers and correlations from this study cannot be generalised to other languages and contexts.

### Response patterns

Findings from earlier studies are reproduced in our material both as of correlation to age and distribution of answering alternatives. A relationship between education and SRH has also been demonstrated in earlier studies [7,11]. In accordance with the semantic analysis, the explanation may lie in stratification, namely that what is compared is social status. This interpretation has semantic support [14 p.225]. What is compared are the sense relations attached to “health” which includes physical functional ability, youthfulness, beauty, habits, lifestyle, food etc. Hence, low SRH does not necessarily express low health in a medical sense; instead it might signal a greater vulnerability to later outbreaks of illness. Low SRH increases the probability of belonging to a group with increased risk for stroke, heart infarction, depression, depressed functioning, and early death. The difference between men and women in answering the Comparative SRH question warrants further investigations.

Our material in 1994 and 1999 revealed a statistically significant increase in the number of people giving the answer “better” to the Comparative SRH question. The placing of the question was changed between the two years (see Results/Context for the questionnaire). The change in the answer distribution might be explained by a health improvement in the population sample. However no similar increase of “better” was observed in the neighbouring county of Västerbotten, where the positioning of the Comparative SRH question remained unchanged in a similar questionnaire. The response rate in the MONICA study fell by 4% between 1994 and 1999. This might have resulted in a shift towards the alternative “better” in 1999. However, the increase remained in the following surveys, indicating that the lower response rate in 1999 is not the main explanation for the shift to the alternative “better”. The results suggest that part of the explanation for the 7 percent unit increase between 1994 and 1999 of the answer “better” was the changed context for the SRH-questions.

### Words and their meaning

Our semantic analysis highlights that the SRH questions are unclear and imprecisely formulated from a scientific point of view, with concepts poorly defined. It may be thought strange for such questions to have found their way into a scientific questionnaire. Their status in the scientific context is defended by their empirically firm link to important health outcomes. Nevertheless, there are theoretical contradictions in how the concept of self-rated health is treated in the scientific literature. Objective health is often regarded as the norm and target for subjective health assessments, while subjective assessments of health are regarded as less accurate rather than different. The correlations of SRH questions with health outcome show that simple everyday speech should not be underestimated as a source of information, in spite of its apparent vagueness.

Health comparisons are frequently made downwards, i.e. with people in worse condition [1,18,19]. The comparison then has the function of supporting and encouraging the person making the comparison. Saying: “Others are worse off, it could be worse” may be a part of a constructive coping strategy. The answers expected from such an interpretation are “it’s better” or “similar”. On the other hand, one might think that some individuals would make the comparison from the perspective “others are better off”, reflecting resignation and a feeling of powerlessness. Epidemiological data supports the theory that social status influences health. The interpretation given by Marmot, and others, is that with higher social status, one has more influence and control over one’s life, which should promote good health [17]. Conversely, those with lower social status have less control over their life situation and are more inclined to despair, leading to a worse health outcome. The Comparative SRH question might be one way to expose this situation.

### Are general SRH and comparative SRH parallel assessments?

Our analysis showed that the General and Comparative SRH questions have semantically different meaning. Consequently one cannot assume that the answers represent parallel judgements of self-rated health. Instead, they are different ways of making an overall health assessment. Similar conclusions were reached by Sargent-Cox et al. in a multiple regression analysis. They state “These results show that the three SRH items are not equivalent measures of health and cannot be used interchangeably” [12].

Vuorisalmi et al. claimed that the age-compared estimate did not measure objective health conditions in the same way for different age groups [20]. They concluded that General SRH was likely to be a more valid measure of general health status and a better predictor of future health than the comparative measure. They asserted that the General SRH question was preferable in a clinical context. Based on our findings we suggest other conclusions. Which question best predicts future health must be decided using appropriately designed studies comparing the predictive quality of the two questions. The Comparative health question provides other information, because a classification, a comparison with others, is required, not merely an adjective. There are reasons to believe that such a classification is significant for health. The answer “worse” might indicate an inferior coping strategy and social position, affecting future health and functioning capability. Finally, we claim that the Comparative SRH question is semantically clearer, because it contains an explicit frame of reference for comparison, and is less disturbed by affective content than the more conversation-based use of the words, “good, bad, in between”.

## Conclusions

Semantic analysis brings insight into how questions of Self-Rated Health could be interpreted.

Health is not assessed primarily in terms of the dictionary denotation of health, but of its sense-relations (associations).

Comparative SRH and General SRH contain different information on SRH, comparative SRH is semantically more distinct but both can be used in questionnaires.

Contexts surrounding the questions in a questionnaire might be or importance for the answers and should therefore be explored in further studies.

Investigate the usefulness of SRH in clinical encounters and its function as a key question.

## Competing interests

The authors declare that they have no competing interests.

## Authors' contributions

GW conceived of the study and wrote the manuscript. PT discussed the semantics and philosophical part and drafted the manuscript. UJ supervised the statistical and epidemiological part and the use of the MONICA-database. KH read several drafts of the manuscript and helped in focusing on more essential parts. AF supervised the project, continually read, criticized and made alternative texts. All authors read and approved the final manuscript.

## Pre-publication history

The pre-publication history for this paper can be accessed here:

http://www.biomedcentral.com/1471-2288/12/154/prepub
